# Sea ice-ocean coupling during Heinrich Stadials in the Atlantic–Arctic gateway

**DOI:** 10.1038/s41598-024-51532-7

**Published:** 2024-01-11

**Authors:** Naima El bani Altuna, Mohamed M. Ezat, Lukas Smik, Francesco Muschitiello, Simon T. Belt, Jochen Knies, Tine L. Rasmussen

**Affiliations:** 1 Department of Geosciences, UiT – The Arctic University of Norway, 9010 Tromsø, Norway; 2 Biogeochemistry Research Centre, School of Geography, Earth and Environmental Sciences, University of Plymouth, Plymouth, PL4 8AA UK; 3 Centre for Resilience in Environment, Water and Waste, College of Life and Environmental Sciences, University of Exeter, Exeter, EX4 4RJ UK; 4 Department of Geography, University of Cambridge, Cambridge, CB2 1BY UK; 5 Geological Survey of Norway, 7040 Trondheim, Norway

**Keywords:** Palaeoceanography, Palaeoclimate

## Abstract

The variability of Arctic sea-ice during abrupt stadial-interstadial shifts in the last glacial period remain poorly understood. Here, we investigated the millennial-scale relationship, with a focus on Heinrich Stadials (HS), between sea-ice cover and bottom water temperature (BWT) during Marine Isotope Stages (MIS) 3 and 2 (64–13 ka) in the Fram Strait using new molecular sea ice biomarker data and published benthic foraminiferal BWT records. Widespread spring sea-ice cover (SpSIC) dominated the studied interval, especially in mid-late MIS 3 (45–29 ka). Yet, warm interstadials were characterized by relatively more open-ocean conditions compared to cold stadials. At the transition between a HS and the subsequent interstadial, sea ice was tightly linked to BWT with rapid reductions in SpSIC coinciding with lower BWT at the end of HS. The relative timing of the events, especially during HS 1, points to ocean warming as the key controlling factor for sea ice reduction at millennial timescales.

Arctic warming is currently causing dramatic reductions in both thickness and extent of sea-ice cover in the Arctic Ocean, which, in turn, is accelerating the impact of climate change at high latitudes^1^. Dansgaard–Oeschger (D–O) events recorded in Greenland ice cores are rapid millennial-scale warming events, which occurred during the last glacial period (c. 115,000 to 11,000 years ago)^2^ at a rate similar to present-day climate warming in the Arctic region^3^. During D-O events, climate oscillated within as little as a few decades from cold (known as Greenland stadials (GSs)) to short-term warm intervals (known as Greenland interstadials (GIs))^2,4,5^. Long-lasting GSs are often associated in North Atlantic sedimentary records with deposition of layers of ice-rafted detritus (IRD), dominance of the polar planktic foraminiferal species *Neogloboquadrina pachyderma*, and low planktic foraminiferal δ^18^O^6^. These IRD layers signify cold Heinrich events in the North Atlantic and the Nordic Seas^6^ caused by a large freshwater supply from melting icebergs released from the Laurentide Ice Sheet^6–8^ and the Greenland, Iceland, and Fennoscandian ice sheets surrounding the Nordic Seas^9^. Records of Heinrich events from the North Atlantic which coincide with stadials are often referred to as Heinrich Stadials (HSs)^10^. Although D-O events have been studied extensively, the exact nature of the mechanisms that drove the abrupt transitions from cold to warm conditions is still under debate. An abrupt reduction in sea-ice cover at the end of GSs and HSs has been proposed as a likely central driver for the subsequent atmospheric rapid warming^11–13^.

Despite the proposed role of sea ice in regulating these millennial-scale cooling and abrupt warming events, the northern extent of the spatial–temporal evolution of sea-ice cover and underlying mechanisms are still poorly resolved by proxy records. The Atlantic Meridional Overturning Circulation (AMOC) regulates the climate of the northern hemisphere by transporting warm and salty Atlantic water (AW) from the tropics to the Arctic and is therefore closely connected to sea-ice dynamics at high northern latitudes^14,15^. During GSs, a weakened AMOC^16,17^, cold atmospheric temperatures, and melting of numerous icebergs created a surface meltwater layer and together contributed to the formation of an extensive sea-ice lid^18–20^. Sea-ice cover and meltwater effectively insulated the warmer subsurface water from the atmosphere, thus limiting heat loss from the ocean, and causing the expansion and deepening of the warm AW to intermediate water depths^21–24^. Under these conditions, a vast subsurface heat reservoir (subsurface temperatures likely warmed up to 5.5 °C^23,24^) accumulated from the mid North Atlantic to the Arctic Ocean beneath the freshwater lid, especially during the GSs associated with Heinrich events^24^. Further, modelling studies have shown that a small temperature increase in subsurface AW^25^, a minor change in the freshwater supply^26^, and/or changes in the wind-stress^12^ potentially triggered the rapid retreat of sea ice at the transition between (cold) GSs and (warm) GIs.

Investigating the anatomy of GIs, GSs and HSs in the Nordic Seas contributes to the understanding of the mechanisms responsible for the D-O events and abrupt oceanic and climatic changes. In this work, we aimed to investigate the interaction and timing of millennial scale changes in sea-ice cover and temperature in the subsurface intermediate AW layer in the Fram Strait, the principal Atlantic–Arctic gateway with a focus on HSs during the late Marine Isotopic Stage (MIS) 4 to MIS 2. Despite some previous biomarker-based sea-ice reconstructions during D-O events in the Nordic Seas^18,19,27^, there have, as yet, been no studies carried out in the Fram Strait beyond 30 ka^28–31^. To address this, we studied piston core HH15-1252PC retrieved at 1257 m water depth from the eastern Fram Strait^24^ and measured the sea-ice biomarker proxy IP_25_ and related highly branched isoprenoid triene (HBI III) that is frequently used as an indicator of the open waters of the Marginal Ice Zone (MIZ) (i.e. HBI III; ref^32^). We also calculated semi-quantitative sea-ice indicators (Spring Sea-Ice Concentration (SpSIC) and a classification tree (CT) model categorizing sea-ice settings; see Material and Methods) along with a recently proposed biomarker proxy for the spring phytoplankton bloom (HBI T_25_^33^). The core is located at c. 79°N in an area that remains seasonally sea-ice free in modern times due to the northward transport of warm Atlantic surface Water^34^ (Fig. 1). Atlantic water (T = 1–4 °C; S = 34.9–35) flows in the uppermost c. 600 m beneath a thin mixed surface water layer^35^. In contrast, in the western Fram Strait, the East Greenland Current (EGC) carries cold, low-saline Polar surface water along with sea ice and icebergs southwards towards Denmark Strait (Fig. 1). Our age-depth model of this high-resolution record (average time resolution between samples 250 years; and a 1-cm sample comprises, on average, 59 years) is based on the correlation between planktic foraminiferal oxygen isotopes and the δ^18^O NGRIP record and is validated further by seven AMS-^14^C dates (Fig. 2A). The record covers the last 64 ka to c. 13 ka, spanning 16 GS-GI transitions and including HS 6 to HS 1^24^. We also compared our new sea-ice reconstruction with published BWT data from the same core^24^ and other sea-ice/BWT records elsewhere in the central and southern Nordic Seas (Table S1).

**Figure 1:**
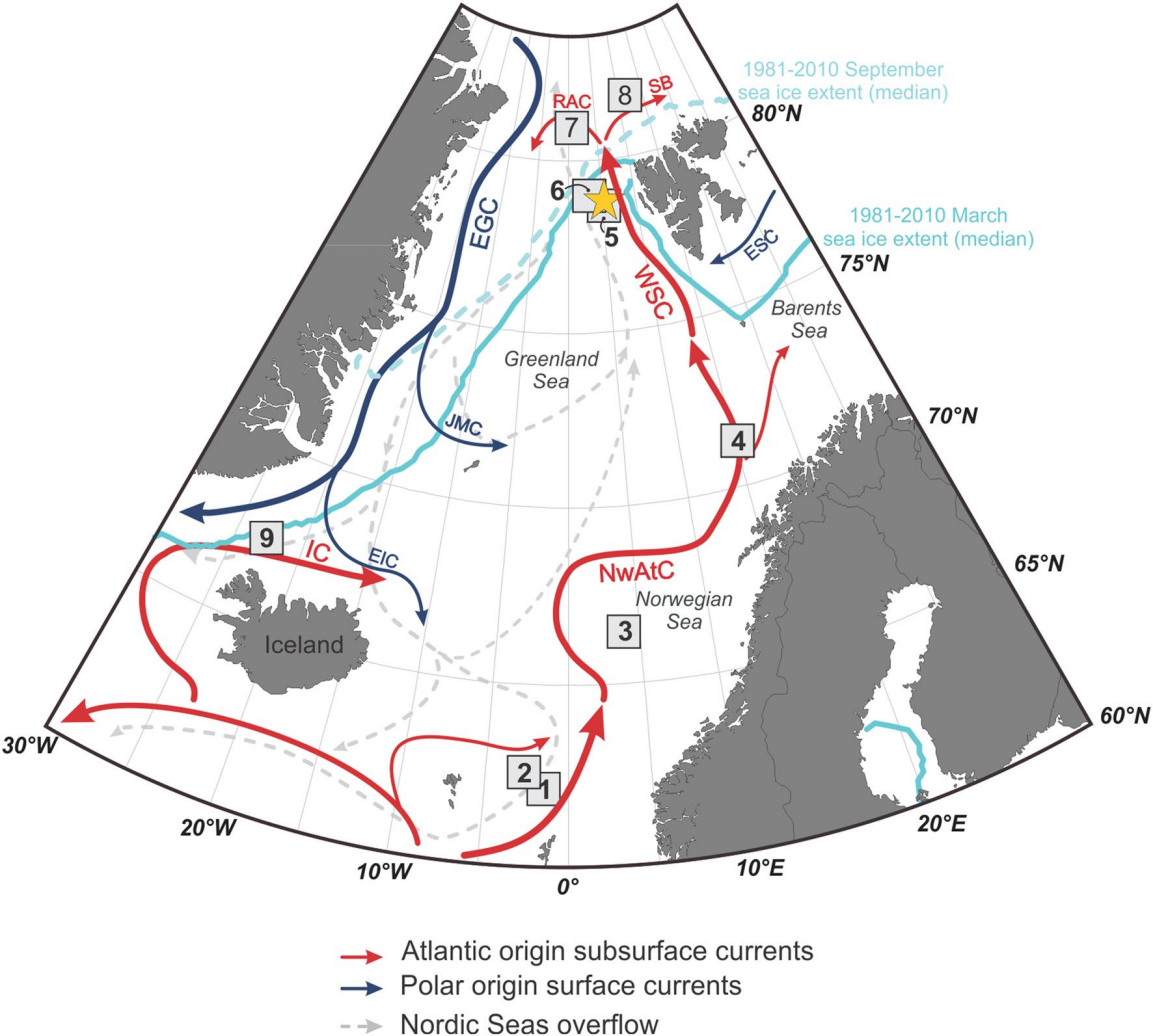
Study area and modern oceanographic setting. Map showing location of core HH15-1252PC on western Svalbard margin (yellow stars) and other cores from literature used in the discussion (gray squares): 1, MD99-2284^19^; 2, JM11-FI-19PC^18^; 3, MD95-2010^20^; 4, GS14-190-01PC^30^; 5, MSM5/5–712-2^29^; 6, PS93/006–1^31^; 7, PS92/039-2^31^; 8, PS2837-5^28^ ; 9, GS15-198-36CC^48^ (see Table S1). Major surface and deep ocean currents and median sea-ice extents for March and September for the period 1981–2010 are also shown (https://nsidc.org/data/seaice_index). *EIC* East Icelandic Current; *EGC* East Greenland Current; *ESC* East Spitsbergen Current; *IC* Iceland Current; *JMC* Jan Mayen Current; *NwAtC* Norwegian Atlantic Current; *RAC* Returning Atlantic Water; *SB* Svalbard Branch; *WSC* West Spitsbergen Current. The map was generated using Ocean Data View (https://odv.awi.de).

**Figure 2:**
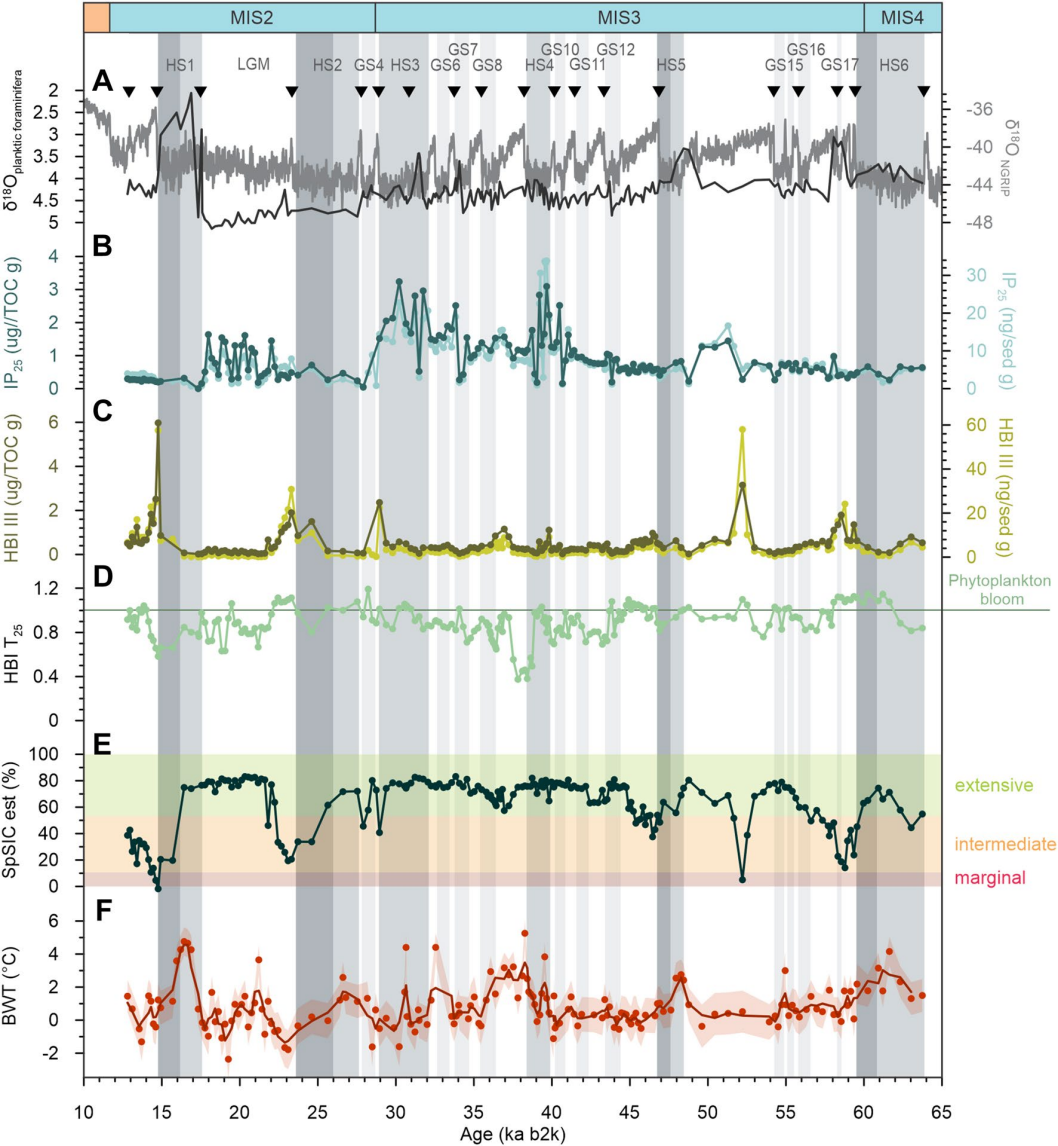
Temporal evolution of sea ice in the western Svalbard margin. (**A**) Planktic foraminiferal (*Neogloboquadrina pachyderma*) δ^18^O^24^ (black line) and NGRIP ice-core δ^18^O on the GICC05modelext b2k timescale^5,60,61^ (grey line), which were used to identify tie-points (inverted triangles) to build the age-depth model for core HH15-1252PC^24^. (**B**) IP_25_ normalized against total organic carbon (dark curve) and sediment weight (light curve). (**C**) HBI III normalized against total organic carbon (dark curve) and sediment weight (light curve). (**D**) HBI T_25_ (values larger than 1 indicate a phytoplankton bloom^33^). (**E**) Relative spring sea-ice concentration (SpSIC). Shaded areas show results from the classification tree, categorizing sea ice into extensive (> 50%; green), intermediate (10–50%; orange) and marginal (< 10%; red). (**F**) Reconstructed bottom water temperature (BWT), using benthic foraminiferal Mg/Ca measured in *Cassidulina neoteretis* and *Melonis barleeanus* and its uncertainty interval (blue shading)^24^. Dark gray shadings mark Heinrich Stadial (HS), with darker grade shades indicating the late stage of HSs when BWT and SpSIC drop. Light gray shadings indicate Greenland Stadial (GS)^24^.

Most notably, our results reveal a concomitant decline in sea-ice concentration and BWT in the Fram Strait during most transitions from HSs to major GIs, suggesting that the reduction in sea ice and the loss of heat from the ocean to the atmosphere are tightly coupled, and probably occurred prior to the abrupt atmospheric warming at the onset of some of the GIs.

## Results

### Sea-ice biomarker data in core HH15-1252PC

The sea-ice proxy IP_25_ is a highly branched isoprenoid (HBI) biomarker produced by certain species of marine Arctic sea-ice diatoms during the spring algal bloom^32,36,37^. Its occurrence in marine sediments is now widely used as a reliable proxy for the presence of seasonal (spring) sea ice in paleo records from polar regions^32,38^. We identified IP_25_ in all samples studied (except for one sample at ca. 17.5 ka), consistent with a near-continuous presence of seasonal sea ice, albeit with variable duration or extent.

During late MIS 4 and early MIS 3 (64–45 ka) IP_25_ values remain generally low, with IP_25_ peaks occurring mostly during the stadials, except for the end of GI 14 (52–49 ka), one of the longest interstadials, when IP_25_ increases. This is preceded by a peak in concentration of HBI III, a molecular biomarker produced by some pelagic diatoms in the open waters of the retreating sea-ice edge or in the marginal ice zone (MIZ)^39^. This may be indicative of a progressive increase in seasonal sea-ice cover after the peak warmth at ca. 52 ka. Highest IP_25_ concentrations occur during the mid-late MIS 3 (45–29 ka), during the Last Glacial Maximum (LGM; 24–19 ka), and the late glacial period (19–17.5 ka) (Fig. 2B).

The high IP_25_ values during the mid-late MIS 3 may have resulted from a gradual retreat of an otherwise extensive sea-ice cover, thus favoring the growth of IP_25_-producing diatoms, or from seasonal sea ice within the dynamic MIZ in the study area. For the same period, the concentrations of HBI III remain generally low (Fig. 2C). The combination of elevated IP_25,_ yet relatively low concentrations of HBI III, suggests the occurrence of a seasonal, but persistent, sea-ice layer in the study area. This interpretation is supported further by estimates of high SpSIC (and supported by the biomarker-based CT model) and low HBI T_25_ indicating only intermittent spring phytoplankton blooms (Fig. 2D,E). The previously published results on the benthic foraminiferal faunas show dominance of the benthic species *Stainforthia* spp. and *Nonionella* spp., which feed on short-lived seasonal pulses of phytoplankton deposited on the sea floor also pointing to presence of an extensive seasonal sea-ice cover^24^ (Fig. S1).

Similarly, the LGM and the late glacial are characterized by substantial shifts between high and low IP_25_ concentrations, accompanied by low/absent HBI III (Fig. 2B,C), likely as a result of rapid fluctuations in the degree of seasonal sea-ice cover. In contrast to the mid-late MIS 3 period, the previously published benthic foraminiferal concentration data for the LGM show maximum concentrations of benthic foraminifera, and assemblages dominated by *Cassidulina neoteretis*^24^, a species that thrives under the influence of cooled Atlantic water and is found in stratified waters with dense sea ice or short-lasting seasonally ice-free conditions (see Cage et al.^40^ and references therein) (Fig. S1).

### Millennial-scale variability in sea ice at the western Svalbard margin

**Figure 3:**
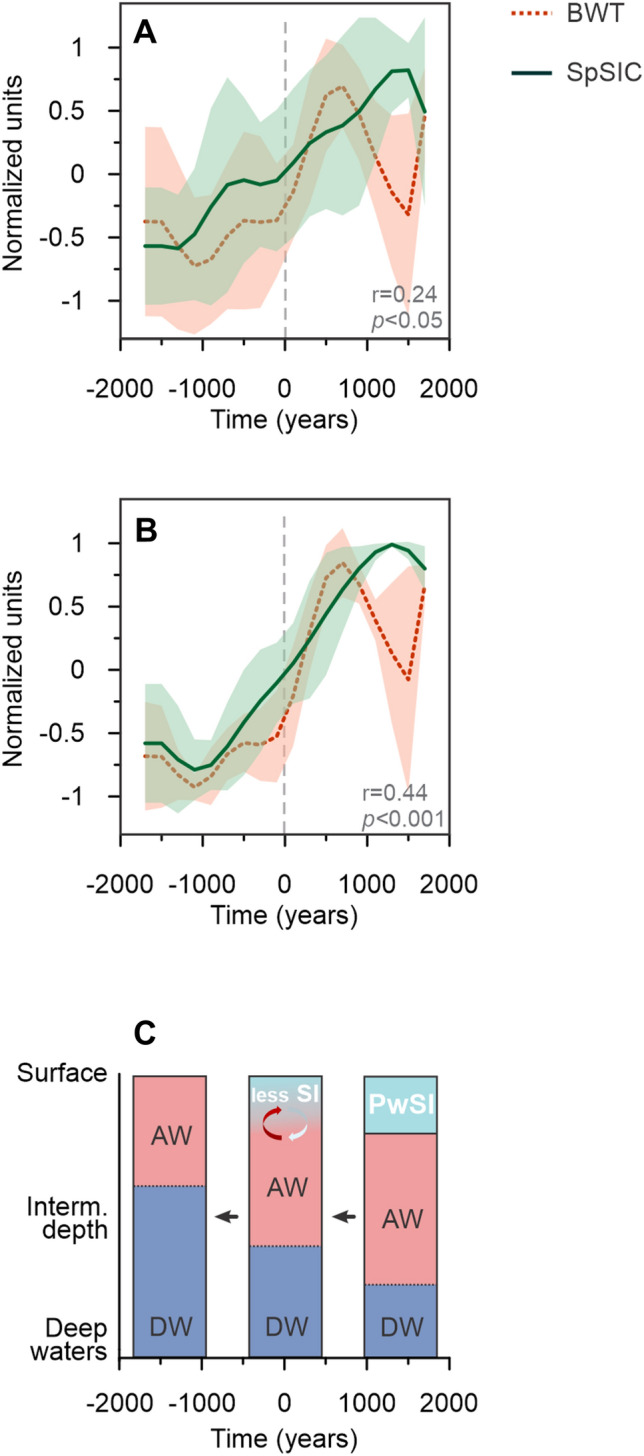
Stacks of Heinrich Stadial (HS) showing normalized bottom water temperature (BWT; red dashed line) and Spring sea-ice concentration (SpSIC; green line) in time. Stack including HS 6 to 1 (**A**), stack including HS 6, HS 5, HS 2, HS 1 (**B**), and the interpreted water column structure for three time slices represented in the stacks (pre-, syn- and post-transition from high to low BWT) (**C**). The thickness of the main water masses is not scaled and is exaggerated. The stacking was done setting time 0 at the mid-point of an abrupt BWT drop during HS (see Material and Methods). *AW* Atlantic Water; *DW* Deep Water; *PwSI* Polar water with Sea Ice; *SI* Sea Ice.

The results from core HH15-1252PC show that seasonal sea-ice (i.e., SpSIC) cover was more extensive than at present during the last glacial period. These long-term severe sea-ice conditions appears to have masked the expected millennial-scale signals in the record. However, a clear millennial-scale pattern can be seen in the distribution of IP_25_ (seasonal sea ice) and HBI III (retreating sea-ice edge/MIZ) for some GSs and GIs (Figs. 2 and S1). The most clearly defined stadials are characterized by generally low IP_25_ (with the exception of events between 30 and 40 ka; see below) and a very low, quasi-absent HBI III, indicating overall extensive sea-ice conditions during most of these periods (Figs. 2B,C and S1). This pattern of low IP_25_ is particularly pronounced for HSs, and especially for HS 6, HS 5, HS 2 and HS 1, when sea-ice cover was likely so significantly advanced and dense that the production of both IP_25_ and HBI III was very restricted. The concentration of IP_25_ increases slightly towards the end of these HSs, as does HBI III, which reaches a maximum at the beginning of the subsequent GI. In addition, SpSIC declines towards the end of each of these HSs. Spring phytoplankton blooms (HBI T_25_ > 1) occurred mainly during GIs, although not exclusively (Fig. 2D). This likely indicates a rapid increase in phytoplankton production due to the close vicinity of the ice margin/MIZ to the core site, and/or longer-lasting seasons of open water concomitant with the initial warming of GIs (Fig. 2C). However, the progressive opening of the ocean surface that started within the HSs (HS 6, HS 5, HS 2 and HS 1) and culminated at the beginning of GIs, suggests that the transition from (1) dense sea-ice cover to (2) a retreating sea-ice edge to (3) open-ocean conditions may not be caused exclusively by the abrupt atmospheric warming, recorded in Greenland ice cores at the beginning of the GIs^5,41^ (Figs. 2 and 3).

Between 30 and 40 ka, HS 4 and HS 3 exhibits higher production of IP_25_ and absence of HBI III, indicating presence of a lasting sea-ice cover (high IP_25_) with only infrequent or transient seasonal open-water conditions at the core site (very low/absence of HBI III). Indeed, conditions may have been similar to the LGM, as indicated by high (but variable) % of *C. neoteretis* (Fig. S1).

### Sea-ice cover and bottom water temperature (BWT) during Heinrich Stadials

Bottom water temperature estimates based on Mg/Ca measurements in benthic foraminifera in core HH15-1252PC^24^ show generally high BWTs (up to 5 °C) during HSs, consistent with studies from the southern Nordic Seas^23,42^. The BWT and sea ice show coherent patterns during most HSs, with the exception of HS 4 and HS 3. We believe this to be due to the greater variability in the BWT data (Figs. 2 and S1) and/or uncertainties in the age model^24^. To investigate the relationship between BWT and sea ice during the HS intervals further, we normalized and stacked our data for all HSs (Figs. 3a; S3A,C; see Material and Methods) and then focused our attention and discussion on HS 6, HS 5, HS 2 and HS 1, while excluding HS 4 and HS 3 (Fig. 3B).

For each of HS 6, HS 5, HS 2, and HS 1, decreases in SpSIC and a transition from extensive to intermediate/marginal sea-ice conditions (Fig. 2) occur alongside reductions in BWT (Figs. 2 and 3), demonstrating concomitant sea-ice retreat and cooling of the intermediate-depth ocean. It is worth noting that non-HS stadials do not show the clear coupling between BWT and SpSIC observed during HSs and have thus not been included in the stacking of data. During HSs, we interpret higher temperatures at deep and intermediate depths in the Fram Strait to be the result of the presence of a strong halocline causing surface stratification, cessation/reduction of deep water formation linked to major AMOC reductions^22^ and a larger southward extent of the sea ice cover in the Nordic Seas^16,18^. This would have reduced the ocean–atmosphere heat exchange and, in turn, allowed warm Atlantic water to flow at greater depths^22–24^. Consistent with these hypotheses, we observe extensive spring sea-ice cover (SpSIC > 50%) at the beginning of HSs during periods with high BWT (Figs. 2E,F and 3). Such conditions would have been unfavorable for both open-ocean and sea-ice related diatom flora, as reflected by low HBI III and IP_25_, respectively (Fig. 2B,C). Although extensive sea-ice conditions dominated during the entire last glacial period, a decline in sea ice from extensive (SpSIC > 50%) to intermediate extent (SpSIC 50–10%) occurred concomitant with reductions in BWT during late HSs (Figs. 2E,F and 3). These results therefore support the presence of a sea-ice lid, which would have limited heat loss from the subsurface to the atmosphere, thus contributing to higher BWT at high latitudes during the early part of HSs^22–24^.

During the major GIs that follow HS 6, HS 5, HS 2 and HS 1, the reactivation of deep water formation could have resulted in generally low BWT, similar to modern day values (ca.  − 0.5–0 °C)^23,24^. In these periods, prominent peaks in HBI III generally occur at the beginning of each GI that follows a HS, are likely the result of a rapid retreat of the sea-ice edge coincident with initial warming (Fig. 2C). Open ocean and reduced sea-ice conditions (relatively higher HBI III) occurred during GIs, although extensive spring sea-ice persisted during the GIs of the cold mid-late MIS 3 and MIS 2 (Fig. 2E).

## Discussion

Our new sea-ice biomarker record from the eastern Fram Strait documents rapid changes in sea-ice cover related to millennial-scale climate oscillations during the last glacial period, most notably during HS 6, HS 5, HS 2 and HS 1 and the corresponding GIs. The Heinrich Stadials HS 4 and HS 3 deviate from this pattern, as they occur within a time interval (45–29 ka) of generally extensive seasonal sea-ice cover that may have masked the millennial timescale signal.

The data reveal two contrasting paleoceanographic scenarios during HSs and the following GIs, with the most extensive sea-ice conditions during HSs, and reduced sea-ice/MIZ conditions during the subsequent GIs (Figs. 2 and S1). The HS to GI transitions are marked by extensive meltwater plumes and release of icebergs from the Svalbard and Barents Sea margin^29,43–45^, which could explain the presence of seasonal sea-ice cover and sea-ice edge/MIZ conditions during the warm GIs. Our results are also in agreement (and partly in disagreement; see discussion below) with sea-ice biomarker data reported previously for the southern and central Nordic Seas^18–20^ with dense sea-ice cover during HS and no or seasonal sea ice cover during the following interstadials.

On longer (orbital) timescales, we observe a more wide-spread and continuous presence of spring sea ice during mid-late (compared to early) MIS 3, similar to a record from the Yermak plateau^31^. This is likely a consequence of a highly stratified water column which favors the formation sea ice^31^. For the LGM, our highly variable biomarker data are also consistent with previous findings from the south-western Barents Sea (c. 71°N)^30^ and from the western Svalbard margin (c. 78°N)^29^, pointing to rapid fluctuations in seasonal sea-ice cover (Figs. S3 and S5). Müller and Stein^29^ interpreted these rapid fluctuations as the result of shifts between perennial and reduced sea-ice cover caused by abrupt changes in the advection of warm and cold AW, which is in line with the recorded BWT at our study site (BWT varying between 4 and  − 2 °C during the LGM^24^; Figs. S4 and S5). Alternatively, Knies et al.^30^ suggested that these fluctuations in seasonal sea-ice extent could be explained by strong katabatic winds blowing offshore from the Svalbard-Barents Sea Ice Sheet, thus creating polynya and upwelling of warmer subsurface water.

**Figure 4:**
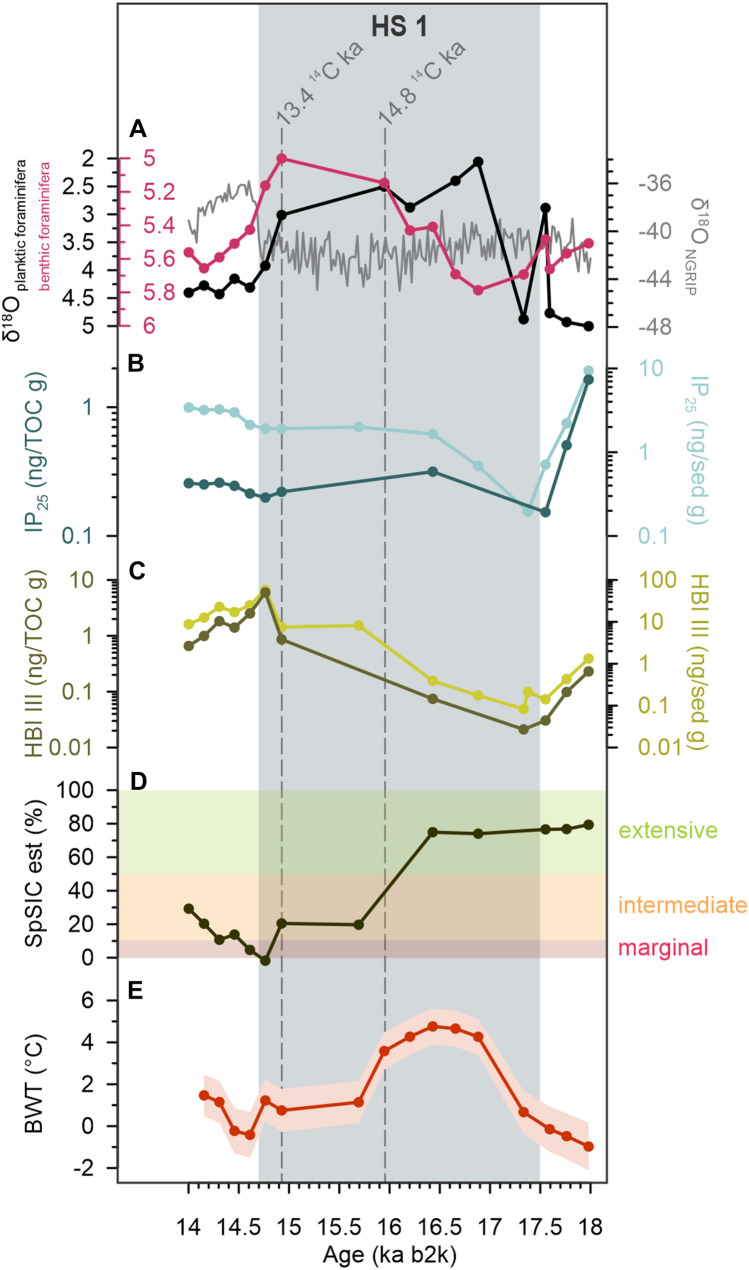
Proxy data in Heinrich Stadial 1. (**A**) NGRIP ice-core δ^18^O on the GICC05modelext b2k timescale^5,60,61^ (gray line) and planktic foraminiferal δ^18^O (δ^18^O_Nps_) (black line). (**B**) IP_25_ normalized against total organic carbon (dark curve) and sediment weight (light curve). (**C**) HBI III normalized against total organic carbon (dark curve) and sediment weight (light curve). (**D**) Relative spring sea-ice concentration (SpSIC). Shaded areas show the results from the classification tree, categorizing the sea ice into extensive (> 50%; green), intermediate (10–50%; orange) and marginal (< 10%; red). (**E**) Reconstructed bottom water temperature (BWT) and its uncertainty interval (blue shading)^24^. Dashed vertical lines mark the depths where radiocarbon dating has been performed; uncalibrated radiocarbon ages shown^24^.

Our data also reveal a co-variability on a millennial timescale between the spring sea-ice cover and BWT during HSs, which suggests a physical coupling between these parameters (Fig. 3). This is best illustrated during Heinrich Stadial 1, with paired BWT and SpSIC data (the sampling resolution being samples every 250–500 years). The timing of HS 1 is relatively well constrained in core HH15-1252PC, both from radiocarbon ages and the large decrease in planktic foraminiferal δ^18^O^24^ (Fig. 4). The decline in BWT and SpSIC during HS 1 (Figs. 2E,F and 4) occurs while planktic foraminiferal δ^18^O values remain low (Fig. 4). HBI III remains nearly absent at the beginning of HS1 and increases slightly after the BWT reaches a maximum (Figs. 2C,F and 4), indicating a progressive change towards open-ocean conditions. Similarly, IP_25_ is also very low/absent at the beginning of HS 1, but then increases slightly at the same time as BWT reaches its maximum (Figs. 2B,F and 4). This points to a scenario with near perennial sea-ice cover that progressively became more seasonal until the BWT reached its maximum. This implies that, in the eastern Fram Strait during HS 1, sea-ice retreat was not initiated by atmospheric temperature increases (as seen in Greenland ice core records) but was also strongly linked to changes in the advection and temperature of the AW, given that the changes in sea ice preceded the initial warming to the GI (i.e. Bølling warming). Sadatzki et al.^19^ likewise observed that changes in sea-ice during HS 4 preceded the initial Greenland warming to GI8.

For the eastern Fram Strait, we show a reduction in sea-ice cover slightly before the end of HSs (HS 6, HS 5 and HS 2) and within HS 1 (Figs. 2 and 3), while in the southeastern Nordic Seas, the decline in sea-ice cover takes place at the end of both GSs and HSs^18,19^ (Figs. S4 and S5; Table S1). Longer-term biomarker-based sea-ice reconstructions from the southeastern Nordic Seas^18^ and the northern Nordic Seas (this work and ref^29^, respectively) exhibit similar trends during HSs (particularly during HS 5, HS 2 and HS 1). In the central Norwegian Sea (Vøring Plateau), a high-resolution study focusing on GSs between 40 and 32 ka (including HS 4) revealed a sea-ice regime change at the transition between some GSs and GIs (e.g. GS 10, GS 9 and GS 8^20^ (Figs. S4 and S5; Table S1). High spring sea-ice concentration in the Nordic Seas is thus consistent north-to-south during HSs, but shows some differences during mid-late MIS 3 and MIS 2, with a clear millennial-scale variability in the south^18–20^ and more persistent sea-ice conditions in the north, probably due to the low insolation and persistent cold conditions at higher latitudes (Figs. 2E and S4; Table S1).

Interestingly, we identify a difference in timing of the reductions in BWT between the Fram Strait and the southern Nordic Seas. In the north^24^, decreases in BWT occurred within stadials, and concomitant with reductions in sea ice (Figs. 3 and 4), while in the south^23,42^ the reduction in BWT occurred by the end of stadials and after the decline in sea-ice cover^18,19^ (Figs. S4 and S5; Table S1). In the eastern Fram Strait, the salinity stratification formed at the onset of stadials would start to weaken because of the presence and build-up of a vast heat reservoir below the halocline^22,24^. This would cause a convective thermohaline instability resulting in the rapid decline of sea ice, formation of open water areas and loss of heat from the surface ocean to the atmosphere (Fig. 3C). Model simulations^46,47^ have shown that in the southern and southeastern waters of Greenland, such thermohaline instabilities led to the formation of polynyas at the end of stadials, which were rapidly followed by major D-O warmings. In the southwestern Nordic Seas, north of Iceland, massive meltwater pooling via the East Icelandic Current could have sustained a (seasonal) sea-ice layer, trapping the AW underneath^9,48^ and delaying the thermohaline instability (i.e. break-up of the stratification), at least during some HSs. Despite differences in time resolution, depositional rates and degrees of bioturbation with latitude, we suggest that some of these geographical variations can be explained by differences in the amount of meltwater input from the SBIS and the Fennoscandian Ice Sheet in the north-eastern and central-eastern (central Norwegian Sea, Vøring Plateau) Nordic Seas (Table S1), compared with (1) the Greenland Ice Sheet via the southeasterly flowing branches of the East Greenland Current (Jan Mayen Current and East Icelandic Current) to the southern Nordic Seas^18,19,48^, and (2) the Celtic and southern Fennoscandian Ice Sheets in the east of the southern Nordic Seas^20,49^ (Fig. 3C). In support of this, the Celtic and the southern Fennoscandian Ice Sheet began retreating during the LGM and in the early HS 1^50,51^, compared to the Svalbard-Barents Sea Ice Sheet, which retreated later during the deglaciation^51–53^. This is supported by an acoustically transparent unit and laminated sediments deposited as the result of a meltwater plume during the LGM and early HS 1 in the southern Nordic Seas^49,54–56^, whereas laminated sediments do not occur in the northern Nordic Seas along the western Svalbard margin until the Bølling-Allerød interstadial^43^.

Our new reconstruction demonstrates a strong coupling between changes in the inflow of subsurface and surface AW in the Fram Strait and sea ice during HSs, with a dense sea-ice cover during HSs and either absent or seasonal sea-ice cover during the following GIs. Additional high-resolution data and chronological improvements are still needed to better constrain the phasing between changes in ocean heat loss and sea-ice variability across the abrupt climate shifts of the last glacial cycle in the Atlantic–Arctic gateway region. However, we observe that for HS 1, at least, both sea ice and BWT decline within the stadial, suggesting that ocean temperature and changes in the sea-ice cover preceded atmospheric warming recorded in Greenland ice cores at the beginning of the GIs and could therefore be one of the contributing forces for such abrupt atmospheric changes. Over longer timescales, our findings indicate that spring sea-ice conditions were widespread throughout the entire last glacial period (Fig. 2). In contrast, BWT fluctuated from modern-like values (during GIs) to exceptionally warm values during HSs both in the northern^24^ and in the southern^23^ Nordic Seas. These results suggest that the coupling of both parameters occurred specifically during most HSs, yet during mid-late MIS 3 (45–29 ka), a generally extended seasonal sea-ice cover in the north may have muted the otherwise relatively clear signals of the GSs and smaller GIs (seen in BWT and distribution of the benthic foraminiferal fauna^24^) compared to the distinct variability in sea/ice biomarker data from the southern Nordic seas^18^.

Regionally, the development of sea-ice cover appears to be tightly related to ocean circulation, atmosphere, and meltwater input from ice sheets, which, in combination, control the stratification of the upper part of the water column and thus the evolution of the sea-ice cover at millennial timescales. The complex configuration of the ocean-cryosphere-atmosphere system and the dynamics of their interactions at millennial timescales during the last glacial period in the Nordic Seas is comparable to that of the modern Arctic Ocean. Based on our observations (i.e. strong linkage between subsurface AW inflow and sea-ice variability) and following the reflections of Aagaard et al.^57^, we can speculate whether the current “Atlantification” in the Barents Sea^58^ and the warming of the atmosphere^59^ will counteract (and even exceed) the increasing freshwater supply from melting ice, thus leading to an even faster disappearance of Arctic sea ice than previously thought.

## Materials and methods

### Core handling

Core HH15-1252PC (79.04°N; 6.89°E; 1257 m water depth; 9.35 m core recovery) was retrieved north of Vestnesa Ridge, on the western Svalbard continental slope on a cruise with the R/V Helmer Hanssen in summer 2015. After retrieval, magnetic susceptibility was measured with a Bartington MS2 loop sensor. The core was subsequently split longitudinally in two halves. The archive halves were X-rayed with a GEOTEK 7.9 Multi Sensor Logger and color imaged with a Jai L-107CC 3 CCD RGB line scan camera installed on an Avaatech XRF at the laboratory of the Department of Geosciences at UiT The Arctic University of Norway in Tromsø. The work halves were subsampled for benthic foraminiferal Mg/Ca analyses to investigate the evolution of bottom water temperature (BWT), as well as benthic and planktic foraminiferal stable isotopes (δ^18^O and δ^13^C), faunal distribution of benthic foraminifera and ice-rafted debris counts (see^24^ for details).

### Radiocarbon dating and age model construction

The age-depth model of core HH15-1252PC was constructed using planktic foraminiferal δ^18^O, supported by the magnetic susceptibility and the down-core distribution of benthic foraminifera *Cassidulina neoteretis*^24^. Planktic foraminiferal δ^18^O from our core were correlated to the North Greenland Ice Project (NGRIP) ice core δ^18^O with the GIC05modelext timescale b2k (i.e. before 2000 AD)^5,60,61^, assuming that massive meltwater events occurred during Greenland Stadials^7^. Thereafter, the age-depth model was built by linear interpolation of the identified tie-points and validated using seven AMS-^14^C dates (see^24^ for details on the construction of the age model).

We used the previously identified tie-points^24^ to build an age-depth model with quantified uncertainties. To account for uncertainties resulting from the spaced sampling intervals (average spacing between adjacent planktic foraminiferal δ^18^O samples of 3.32 cm) and the visual identification of tie-points, we used a fixed uncertainty around the tie-points of 150 years following the approach by Hughen and Heaton^62^. The uncertainties for each tie-point were then used to build the age-depth model using the Bayesian-based package BACON^63^ in R Software.

Based on the new age model, core HH15-1252PC spans from 63.8 to 12.9 ka, covering Marine Isotopic Stages (MIS) 4 to MIS 2.

### Biomarker analysis

For biomarker analyses, a total of 201 samples were subsampled from the archive halves of the core in 1-cm thick slices in intervals of 1 to 5 cm (one sample subsampled at a 7-cm interval), with higher resolution sampling carried out for intervals with high BWT. Samples were freeze dried, homogenized, and stored in glass vials.

Lipid analysis was carried out according to Belt et al.^64^, but with a slight modification to the extraction method. Thus, freeze-dried samples (ca. 5 g) were saponified in a methanolic Potassium Hydroxide (KOH) solution (ca. 10 mL Methanol:MilliQ water (9:1); 5% KOH) for 60 min (70 °C). Hexane (3 × 2 mL) was added to the cooled (room temperature) saponified content, with non-saponifiable lipids (NSLs) transferred to clean vials and dried over Nitrogen (N_2_, 25 °C). NSLs were then further fractionated using silica (SiO_2_; 0.5 g) column chromatography with non-polar fractions containing HBIs eluted with hexane (6 mL). Prior to extraction, samples were spiked with an internal standard (9-octylheptadec-8-ene, 9-OHD, 100 ng) to permit quantification.

Analysis of purified fractions containing HBIs was carried out using an Agilent 7890A GC coupled to a 5975 series mass selective detector (MSD) and operating conditions specified in Belt et al.^64^. The identification of individual HBIs was based on their characteristic retention indices and mass spectra (Belt, 2018), while quantification was achieved by comparison of mass spectral responses of selected ions (*m/z* 350 (IP_25_); 348 (HBI II, also referred to as IPSO_25_^32^); 346 (HBI III and IV)) with those of internal standard (9-OHD, *m/z* 350), and normalized according to their respected instrumental response factors and the mass of sediment extracted^64^.

Semi-quantitative measures of spring sea-ice concentrations (SpSIC) and HBI T_25_ index (a measure of phytoplankton blooms at the productive marginal ice zone) were derived according the Eqs. (1) and (2) respectively^33,65^.

1 $$SpSIC\left( \% \right) = \left( {\left( {\frac{{IP_{25} }}{{\left( {\left( {IP_{25} + HBI III} \right) \times 0.63} \right)}}} \right) - 0.0692} \right)/0.0107$$

2 $$T_{25} = \frac{{\left( {\frac{HBI III}{{\left( {HBI III + HBI IV} \right)}}} \right)}}{0.62}$$

Classification tree (CT) methods^66^ were used to further categories spring sea-ice conditions into marginal (0–10%), intermediate (10–50%), and extensive (> 50%).

### Determination of total carbon (TC) and total organic carbon (TOC)

To measure the total organic carbon (TOC) ca. 3 g of sediment was subsampled every 5 cm. Dried and powdered samples were treated with 10% hydrochloric acid (HCl) for the removal of carbonates and subsequently measured in a Leco CS-200 at Department of Geosciences at UiT the Arctic University of Norway in Tromsø.

Due to differing sampling intervals in the biomarker analysis and the organic carbon analyses, 33 samples do not have paired carbon content measurements. However, the downcore trends and relative changes of lipid biomarker data normalized with the bulk sediment weight and with the total organic content do not show any deviation from each other (Fig. 2B) and therefore it was deemed unnecessary to carry out such analyses in the remaining samples.

### Statistical analysis

To construct the stacks presented in Figs. 3 and S3 we used an approach that consist of the following steps. First, we estimated the timing of significant changes in BWT throughout the record by applying a Bayesian change point analysis method using the ‘bcp’ package in R^67^. We detected changes in the mean values of the BWT timeseries that crossed a credibility level of 95%. Using visual inspection of the results, we then narrowed down the number of change points to those corresponding to Heinrich Stadials, which for our data set sums up to six transitions (i.e. HS 6 to HS 1; Fig. S3A–C). Next, we defined a time vector *t* that spans from -1500 to + 1500 years at 200-year steps. We set each of the six transitions defined in the BWT data to *t* = 0, and linearly interpolated the different records (i.e. BWT, HBI III, IP_25_, SpSIC) onto the time vector *t*. The interpolation step was chosen based on the mean temporal resolution across all the proxy records considered for stacking. Finally, with all the individual events resampled to identical time spacing, we estimated the correlation (Figs. 3 and S3) and then scaled the data between  − 1 and 1, and averaged them to obtain stacked records. The resulting stacks, excluding HS 4 and HS 3 (Fig. S3D–F; see main text), were used to examine the phasing and co-variability between BWT and sea-ice proxies relative to the transitions observed in the BWT data.

### Supplementary Information

Supplementary Information.

## Data Availability

The data is available at the UiT Open Research Data repository: https://doi.org/10.18710/Z0ODIA

## References

[1] Dai A, Luo D, Song M, Liu J (2019). Arctic amplification is caused by sea-ice loss under increasing CO_2_. Nature Communications.

[2] Dansgaard W (1982). A new Greenland deep ice core. Science.

[3] Jansen E (2020). Past perspectives on the present era of abrupt Arctic climate change. Nature Climate Change.

[4] Johnsen SJ (1992). Irregular glacial interstadials recorded in a new Greenland ice core. Nature.

[5] Rasmussen SO (2014). A stratigraphic framework for abrupt climatic changes during the Last Glacial period based on three synchronized Greenland ice-core records: Refining and extending the INTIMATE event stratigraphy. Quat. Sci. Rev..

[6] Heinrich H (1988). Origin and consequences of cyclic ice rafting in the northeast Atlantic Ocean during the past 130,000 years. Quat. Res..

[7] Bond G (1993). Correlations between climate records from North Atlantic sediments and Greenland ice. Nature.

[8] Barker S (2015). Icebergs not the trigger for North Atlantic cold events. Nature.

[9] Lekens WAH, Sejrup HP, Haflidason H, Knies J, Richter T (2006). Meltwater and ice rafting in the southern Norwegian Sea between 20 and 40 calendar kyr B.P: Implications for Fennoscandian Heinrich events. Paleoceanography.

[10] Barker S (2009). Interhemispheric Atlantic seesaw response during the last deglaciation. Nature.

[11] Gildor H, Tziperman E (2003). Sea-ice switches and abrupt climate change. Philos. Trans. R. Soc. London Series A: Math. Phys. Eng. Sci..

[12] Li C, Battisti DS, Bitz CM (2010). Can North Atlantic sea ice anomalies account for Dansgaard–Oeschger climate signals?. Journal of Climate.

[13] Li C, Born A (2019). Coupled atmosphere-ice-ocean dynamics in Dansgaard-Oeschger events. Quat. Sci. Rev..

[14] Aagaard K, Foldvik A, Hillman SR (1987). The west spitsbergen current: Disposition and water mass transformation. Journal of Geophysical Research.

[15] Ivanov VV, Alexeev VA, Repina I, Koldunov NV, Smirnov A (2012). Tracing Atlantic water signature in the Arctic sea ice cover east of Svalbard. Adv. Meteorol..

[16] Henry LG (2016). North Atlantic ocean circulation and abrupt climate change during the last glaciation. Science.

[17] Lynch-Stieglitz J (2017). The Atlantic meridional overturning circulation and abrupt climate change. Annu. Rev. Mar. Sci..

[18] Hoff U, Rasmussen TL, Stein R, Ezat MM, Fahl K (2016). Sea ice and millennial-scale climate variability in the Nordic seas 90 kyr ago to present. Nature Communications.

[19] Sadatzki H (2019). Sea ice variability in the southern Norwegian Sea during glacial Dansgaard-Oeschger climate cycles. Science Advances.

[20] Sadatzki H (2020). Rapid reductions and millennial-scale variability in Nordic Seas sea ice cover during abrupt glacial climate changes. Proceedings of the National Academy of Sciences.

[21] Rasmussen TL, Thomsen E, Labeyrie L (1996). Circulation changes in the Faeroe-Shetland Channel correlating with cold events during the last glacial period (58–10 ka). Geology.

[22] Rasmussen TL, Thomsen E (2004). The role of the North Atlantic Drift in the millennial timescale glacial climate fluctuations. Palaeogeography, Palaeoclimatology, Palaeoecology.

[23] Ezat MM, Rasmussen TL, Groeneveld J (2014). Persistent intermediate water warming during cold stadials in the southeastern Nordic seas during the past 65 k.y. Geology.

[24] El bani Altuna N, Ezat MM, Greaves M, Rasmussen TL (2021). Millennial-scale changes in bottom water temperature and water mass exchange through the Fram Strait 79^o^ N, 63–13 ka. Paleoceanogr. Paleoclimatol..

[25] Jensen MF, Nisancioglu KH, Spall MA (2018). Large changes in sea ice triggered by small changes in atlantic water temperature. J. Climate.

[26] Jensen MF, Nilsson J, Nisancioglu KH (2016). The interaction between sea ice and salinity-dominated ocean circulation: implications for halocline stability and rapid changes of sea ice cover. Climate Dynamics.

[27] Wary M (2017). The southern Norwegian Sea during the last 45 ka: hydrographical reorganizations under changing ice-sheet dynamics. Journal of Quaternary Science.

[28] Müller J, Massé G, Stein R, Belt ST (2009). Variability of sea-ice conditions in the Fram Strait over the past 30,000 years. Nature Geosci..

[29] Müller J, Stein R (2014). High-resolution record of late glacial and deglacial sea ice changes in Fram Strait corroborates ice–ocean interactions during abrupt climate shifts. Earth and Planetary Science Letters.

[30] Knies J (2018). Nordic Seas polynyas and their role in preconditioning marine productivity during the Last Glacial Maximum. Nature Communications.

[31] Kremer A (2018). Changes in sea ice cover and ice sheet extent at the Yermak Plateau during the last 160 ka: Reconstructions from biomarker records. Quat. Sci. Rev..

[32] Belt ST (2018). Source-specific biomarkers as proxies for Arctic and Antarctic sea ice. Organic Geochemistry.

[33] Belt ST, Smik L, Köseoğlu D, Knies J, Husum K (2019). A novel biomarker-based proxy for the spring phytoplankton bloom in Arctic and sub-arctic settings – HBI T_25_. Earth and Planetary Science Letters.

[34] Hopkins TS (1991). The GIN Sea—A synthesis of its physical oceanography and literature review 1972–1985. Earth Science Reviews.

[35] Ferré B, Mienert J, Feseker T (2012). Ocean temperature variability for the past 60 years on the Norwegian-Svalbard margin influences gas hydrate stability on human time scales. Journal of Geophysical Research.

[36] Belt ST (2007). A novel chemical fossil of palaeo sea ice: IP_25_. Organic Geochemistry.

[37] Brown TA, Belt ST, Tatarek A, Mundy CJ (2014). Source identification of the Arctic sea ice proxy IP25. Nature Communications.

[38] Belt ST, Müller J (2013). The Arctic sea ice biomarker IP_25_: A review of current understanding, recommendations for future research and applications in palaeo sea ice reconstructions. Quat. Sci. Rev..

[39] Belt ST (2015). Identification of paleo Arctic winter sea ice limits and the marginal ice zone: Optimised biomarker-based reconstructions of late Quaternary Arctic sea ice. Earth and Planetary Science Letters.

[40] Cage AG, Pieńkowski AJ, Jennings A, Knudsen KL, Seidenkrantz M-S (2021). Comparative analysis of six common foraminiferal species of the genera *Cassidulina*, *Paracassidulina* and *Islandiella* from the Arctic-North Atlantic domain. J. Micropalaeontol..

[41] Kindler P (2014). Temperature reconstruction from 10 to 120 kyr b2k from the NGRIP ice core. Clim. Past..

[42] Sessford EG (2019). Consistent fluctuations in intermediate water temperature off the coast of Greenland and Norway during Dansgaard-Oeschger events. Quat. Sci. Rev..

[43] Jessen SP, Rasmussen TL, Nielsen T, Solheim A (2010). A new Late Weichselian and Holocene marine chronology for the western Svalbard slope 30,000–0 cal years BP. Quat. Sci. Rev..

[44] Rasmussen TL, Thomsen E (2013). Pink marine sediments reveal rapid ice melt and Arctic meltwater discharge during Dansgaard-Oeschger warmings. Nature Communications.

[45] Jessen SP, Rasmussen TL (2019). Ice-rafting patterns on the western Svalbard slope 74–0 ka: Interplay between ice-sheet activity, climate and ocean circulation. Boreas.

[46] Peltier WR, Vettoretti G (2014). Dansgaard-Oeschger oscillations predicted in a comprehensive model of glacial climate: A “kicked” salt oscillator in the Atlantic. Geophysical Research Letters.

[47] Vettoretti G, Peltier WR (2016). Thermohaline instability and the formation of glacial North Atlantic super polynyas at the onset of Dansgaard–Oeschger warming events. Geophysical Research Letters.

[48] Sessford EG (2018). High-resolution benthic Mg/ca temperature record of the intermediate water in the Denmark strait across D–O stadial-interstadial cycles. Paleoceanogr. Paleoclimatol..

[49] Becker LWM, Sejrup HP, Hjelstuen BO, Haflidason H, Dokken TM (2018). Ocean-ice sheet interaction along the SE Nordic Seas margin from 35 to 15 ka BP. Marine Geology.

[50] Toucanne S (2015). Millennial-scale fluctuations of the European Ice Sheet at the end of the last glacial, and their potential impact on global climate. Quat. Sci. Rev..

[51] Sejrup HP (2022). The role of ocean and atmospheric dynamics in the marine-based collapse of the last Eurasian Ice Sheet. Commun. Earth Environ..

[52] Hughes ALC, Gyllencreutz R, Lohne ØS, Mangerud J, Svendsen JI (2016). The last Eurasian ice sheets: A chronological database and time-slice reconstruction, DATED-1. Boreas.

[53] Patton H (2017). Deglaciation of the Eurasian ice sheet complex. Quat. Sci. Rev..

[54] Hjelstuen B (2004). Late Quaternary seismic stratigraphy and geological development of the south Vøring margin, Norwegian Sea. Quat. Sci. Rev..

[55] Hjelstuen BO (2005). Late Cenozoic glacial history and evolution of the Storegga Slide area and adjacent slide flank regions, Norwegian Continental margin. Marine and Petroleum Geology.

[56] Lekens WAH (2005). Laminated sediments preceding Heinrich event 1 in the Northern North Sea and Southern Norwegian Sea: Origin, processes and regional linkage. Marine Geology.

[57] Aagaard K, Coachman LK, Carmack E (1981). On the halocline of the Arctic Ocean. Deep Sea Res. Part A Oceanogr. Res. Pap..

[58] Asbjørnsen H, Årthun M, Skagseth Ø, Eldevik T (2020). Mechanisms underlying recent arctic atlantification. Geophysical Research Letters.

[59] IPCC. *Climate Change 2021: The Physical Science Basis. Contribution of Working Group I to the Sixth Assessment Report of the Intergovernmental Panel on Climate Change*. (Cambridge University Press, 2021).

[60] Svensson A (2008). A 60 000 year Greenland stratigraphic ice core chronology. Clim. Past..

[61] Wolff EW, Chappellaz J, Blunier T, Rasmussen SO, Svensson A (2010). Millennial-scale variability during the last glacial: The ice core record. Quat. Sci. Rev..

[62] Hughen KA, Heaton TJ (2020). Updated cariaco basin ^14^ C calibration dataset from 0–60 cal kyr BP. Radiocarbon.

[63] Blaauw M, Christen JA (2011). Flexible paleoclimate age-depth models using an autoregressive gamma process. Bayesian Analysis.

[64] Belt ST (2012). A reproducible method for the extraction, identification and quantification of the Arctic sea ice proxy IP_25_ from marine sediments. Analytical Methods.

[65] Smik L, Cabedo-Sanz P, Belt ST (2016). Semi-quantitative estimates of paleo Arctic sea ice concentration based on source-specific highly branched isoprenoid alkenes: A further development of the PIP_25_ index. Organic Geochemistry.

[66] Köseoğlu D (2018). Complementary biomarker-based methods for characterising Arctic sea ice conditions: A case study comparison between multivariate analysis and the PIP_25_ index. Geochimica et Cosmochimica Acta.

[67] Erdman C, Emerson JW (2007). bcp: An R Package for Performing a Bayesian Analysis of Change Point Problems. J. Stat. Soft..

