# Publisher Correction: Sea ice-ocean coupling during Heinrich Stadials in the Atlantic–Arctic gateway

**DOI:** 10.1038/s41598-024-54224-4

**Published:** 2024-02-14

**Authors:** Naima El bani Altuna, Mohamed M. Ezat, Lukas Smik, Francesco Muschitiello, Simon T. Belt, Jochen Knies, Tine L. Rasmussen

**Affiliations:** 1 Department of Geosciences, UiT – The Arctic University of Norway, 9010 Tromsø, Norway; 2 Biogeochemistry Research Centre, School of Geography, Earth and Environmental Sciences, University of Plymouth, Plymouth, PL4 8AA UK; 3 Centre for Resilience in Environment, Water and Waste, College of Life and Environmental Sciences, University of Exeter, Exeter, EX4 4QF UK; 4 Department of Geography, University of Cambridge, Cambridge, CB2 1BY UK; 5 Geological Survey of Norway, 7040 Trondheim, Norway

Correction to: *Scientific Reports* 10.1038/s41598-024-51532-7, published online 11 January 2024

In the original version of this Article Simon T. Belt was incorrectly affiliated with ‘Geological Survey of Norway, 7040, Trondheim, Norway’. Their correct affiliation is listed below.

Biogeochemistry Research Centre, School of Geography, Earth and Environmental Sciences, University of Plymouth, Plymouth, PL4 8AA, UK.

The original Article and accompanying Supplementary Information file have been corrected.

