# Supplementary material for: Sea ice-ocean coupling during Heinrich Stadials in the Atlantic–Arctic gateway
Source: Sci Rep. 2024 Jan 11;14:1065. doi: 10.1038/s41598-024-51532-7 (PMC10784495; doi:10.1038/s41598-024-51532-7)
Supplement: Supplementary file 1 — Supplementary Information. [file 41598_2024_51532_MOESM1_ESM.docx]

**Supporting Information for:**

**Sea ice-ocean coupling during Heinrich Stadials in the Atlantic-Arctic gateway**

Naima El bani Altuna^1,*^, Mohamed M. Ezat^1^, Lukas Smik^2,3^, Francesco Muschitiello^4^, Simon T. Belt^2^, Jochen Knies^5^, Tine L. Rasmussen^1^

^1^Department of Geosciences, UiT – The Arctic University of Norway, 9010 Tromsø, Norway.

^2^Biogeochemistry Research Centre, School of Geography, Earth and Environmental Sciences, University of Plymouth, Plymouth, PL4 8AA, UK.

^3^Centre for Resilience in Environment, Water and Waste, College of Life and Environmental Sciences, University of Exeter, Exeter, EX4 4QF, UK.

^4^Department of Geography, University of Cambridge, Cambridge, CB2 1BY, UK.

^5^Geological Survey of Norway, Trondheim, 7040, Norway.

*Corresponding author: Naima El bani Altuna, [naima.elbani.altuna@uit.no](mailto:naima.elbani.altuna@uit.no)

**This PDF file includes:**

Supporting text

Figures S1 to S5

Tables S1 to S2

SI References

Supporting Information Text

**Correlation with published sea-ice records**

Core HH15-1252PC was tuned to the GICC05modelext timescale b2k (1). To allow comparison between our sea-ice biomarker record and other records in the Nordic Seas, we correlated core HH15-1252PC to other cores (Table S1) using the planktic foraminiferal δ^18^O maxima and minima (Fig. S4B and Fig. S5B), when their chronology was originally based on radiocarbon dating. With cores that were tuned to NGRIP, we used their originally published age-depth model (Table S1).

**
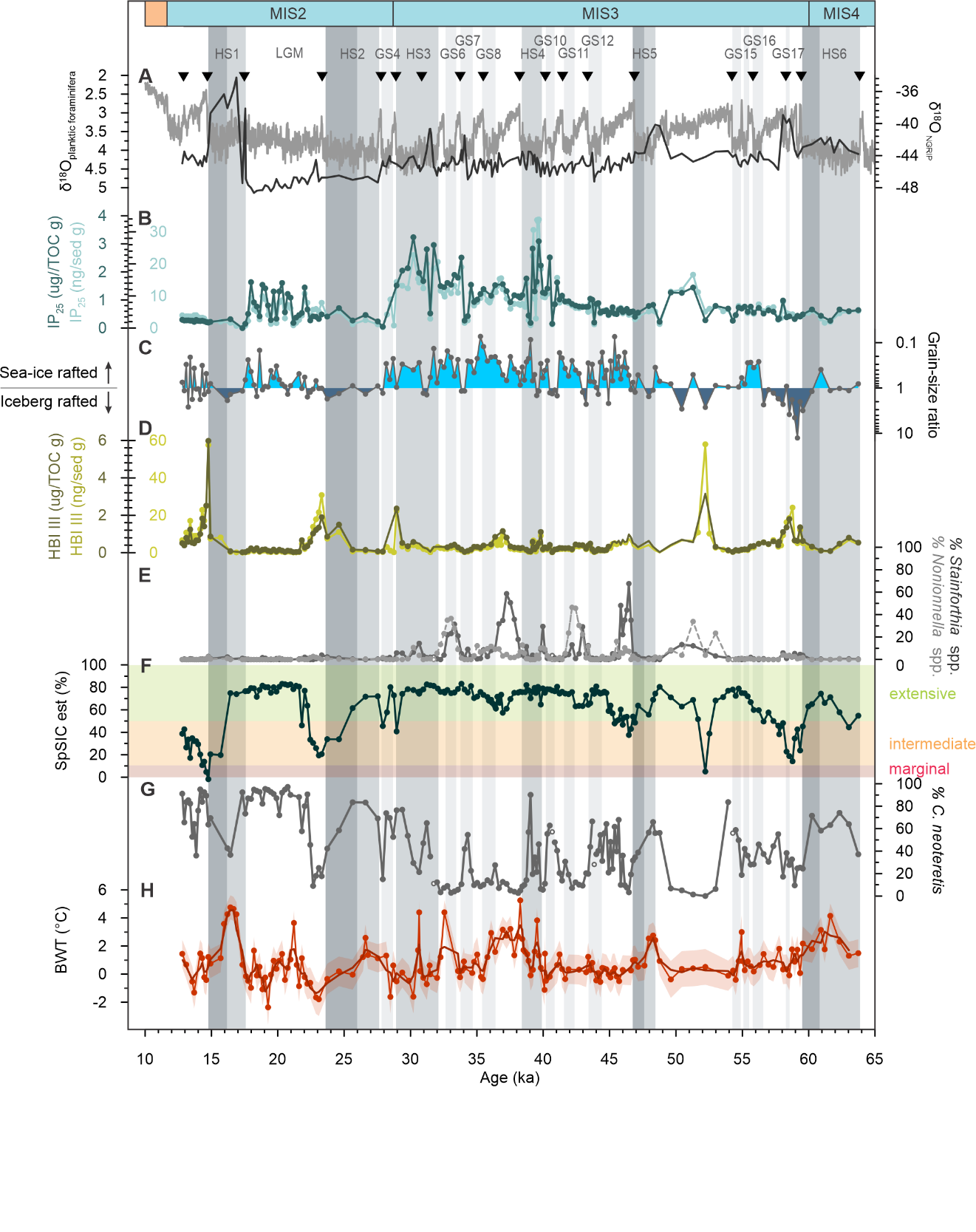
Fig. S1. Temporal variability of benthic foraminiferal species distribution and sea ice data from core HH15-1252PC. (A)** NGRIP ice-core δ^18^O on the GICC05modelext b2k timescale (2–4), which was used to build the age-depth model for core HH15-1252PC (1). **(B)** IP_25_ normalized against total organic carbon (dark curve) and sediment weight (light curve). **(C)** Ice rafted debris grain-size ratio (150–500 μm/ >500 μm) calculated following Jessen et al. (5); values >1 indicate iceberg rafted debris and <1 indicate sea-ice rafted debris. **(D)** HBI III normalized against total organic carbon (dark curve) and sediment weight (light curve). **(E)** Relative abundance of benthic foraminifera *Stainforthia* spp. and *Nonionella* spp. (1). **(F)** Relative spring sea-ice concentration (SpSIC). Shaded areas show results from the classification tree, categorizing sea ice into extensive (>50%; green), intermediate (10–50%; orange) and marginal (<10%; red). **(G)** Relative abundance of benthic foraminifera *Cassidulina neoteretis* (1)*.* **(H)** Reconstructed bottom water temperature (BWT) and its uncertainty interval (red shading) (1). Dark gray shadings mark Heinrich Stadial (HS), with darker grade shades indicating the late stage of HSs when BWT and SpSIC drop. Light gray shadings indicate Greenland Stadial (GS) (1).


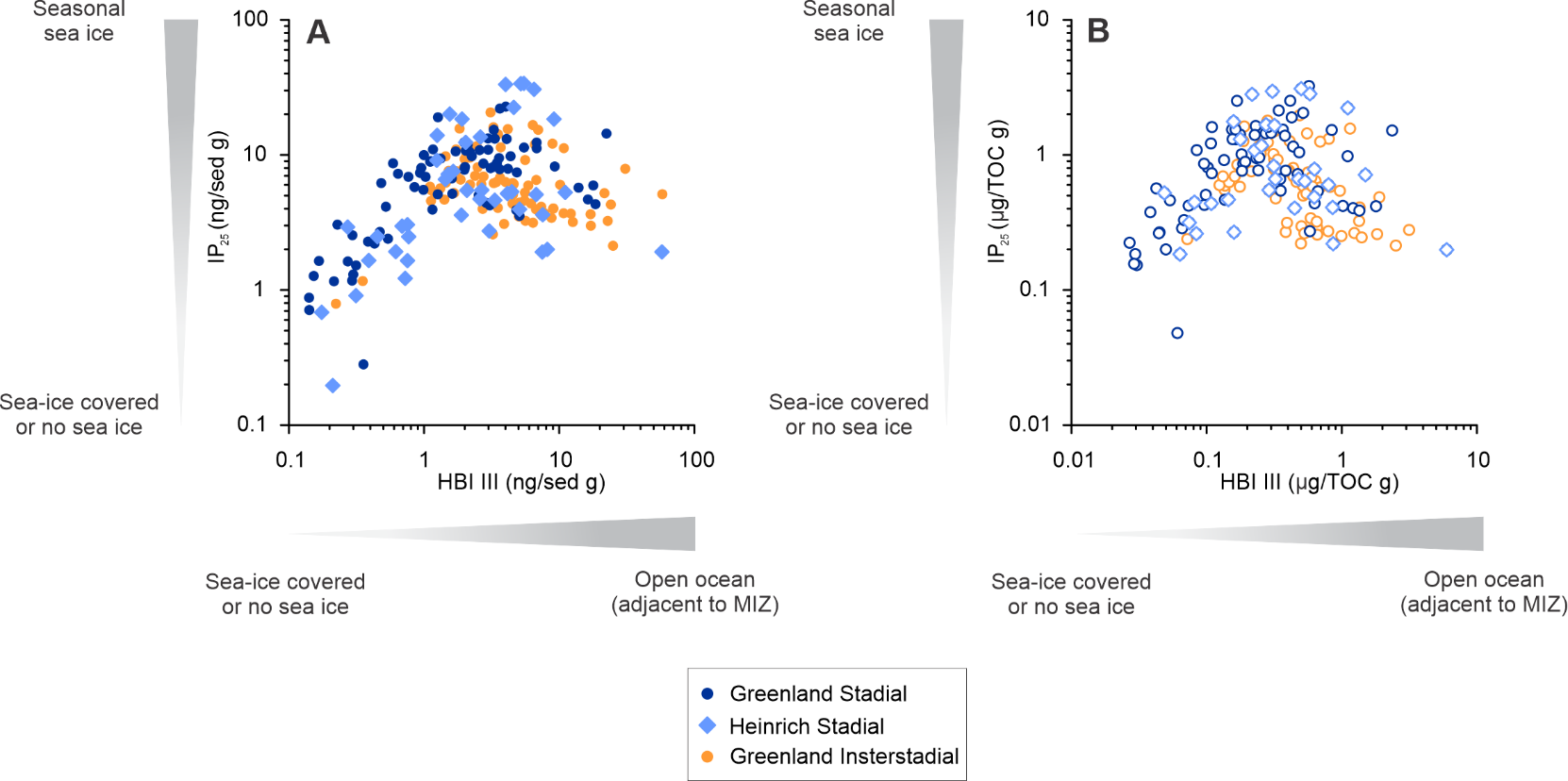
Fig. S2. Cross-plot of IP_25_ and HBI III in core HH15-1252PC. (A) IP_25_/sediment g and HBI III/sediment g and (B) IP_25_/TOC g and HBI III/TOC g have been classified chronologically into Greenland Stadial (dark blue circle), Heinrich Stadial (light blue diamond) Greenland Interstadial (orange circle).


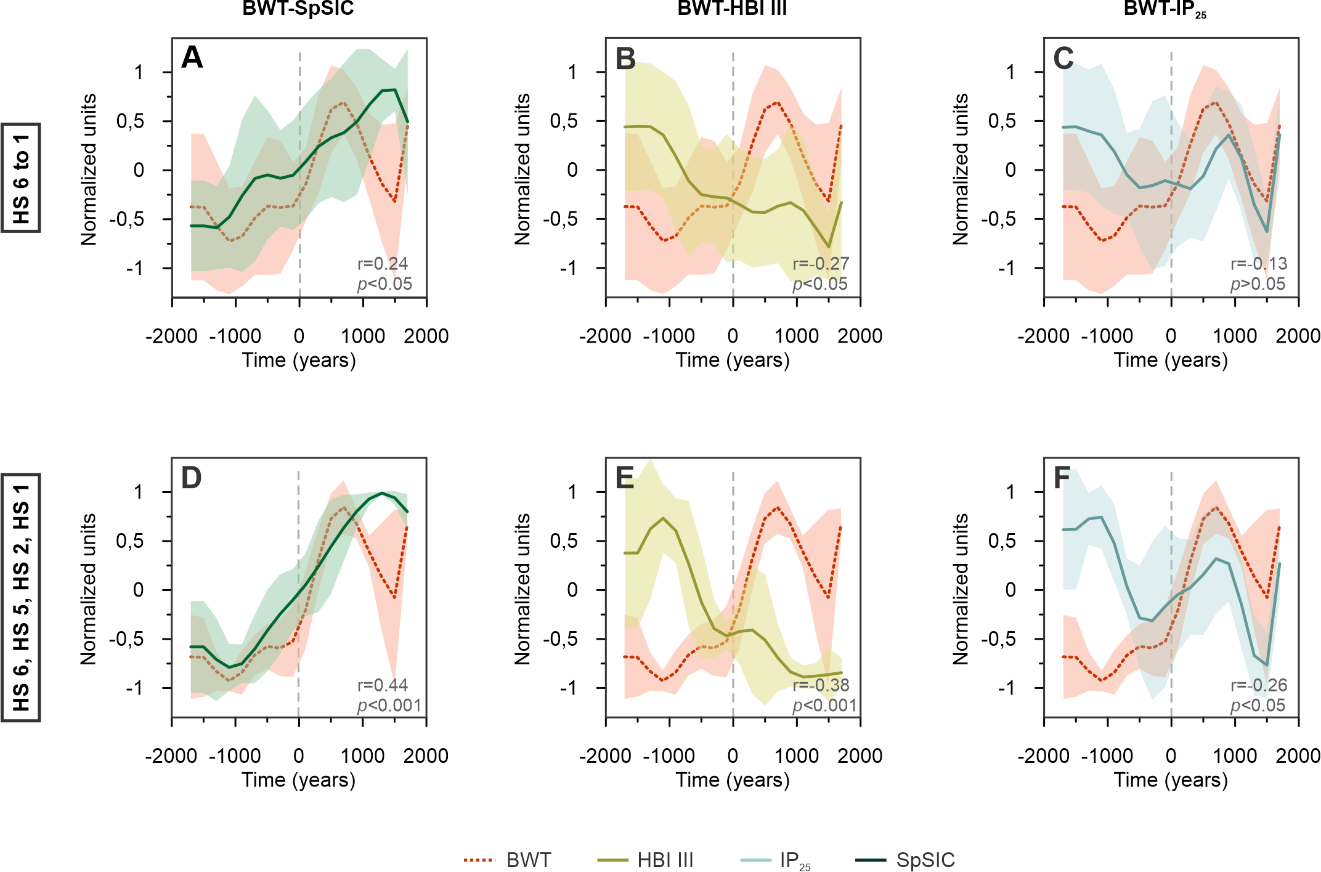
Fig. S3. Stacks of all Heinrich Stadial (HS) in core HH15-1252PC showing normalized bottom water temperature (BWT) and sea-ice variability in time. Stacks including HS 6 to 1 (A, B, C) and stacks including HS 6, HS 5, HS 2, HS 1 (D, E, F). Stacking was done setting time 0 at the mid-point of an abrupt BWT drop during HS (see Material and Methods).

**
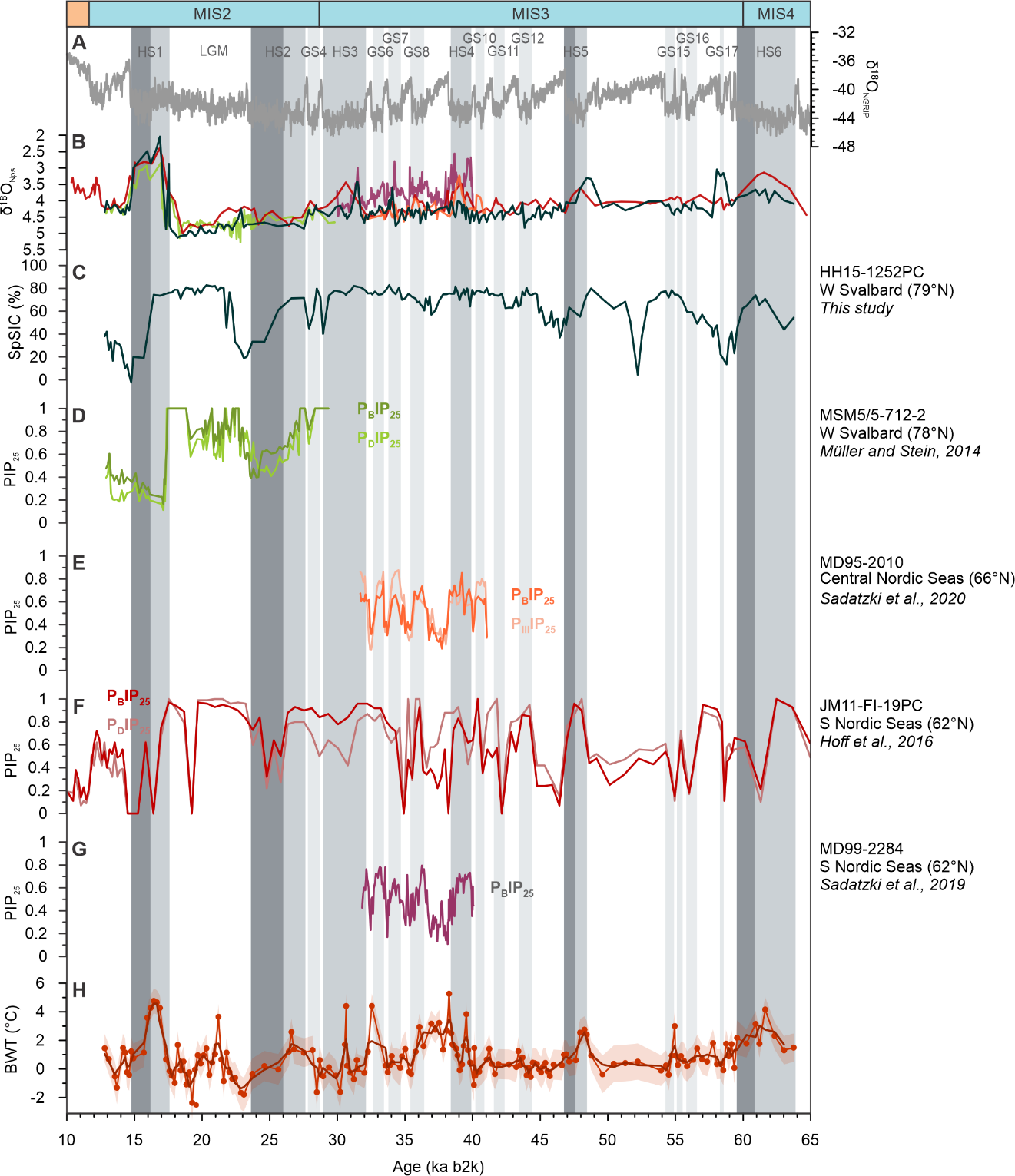
**

**Fig. S4. Compilation of PIP_25_ data from the south-east Nordic Seas and spring sea-ice concentration (SpSIC) in the northern Nordic Seas.**(**A**) NGRIP ice-core δ^18^O on the GICC05modelext b2k timescale (2–4). (**B**) Planktic foraminiferal δ^18^O (δ^18^O_Nps_) of the different cores. (**C**) SpSIC of core HH15-1252PC. (**D–G**) PIP_25_recorded in cores from literature and used in discussion (see Table S1). (**J**) Reconstructed bottom water temperature (BWT) and its uncertainty interval (red shading) (1). Dark gray shadings mark Heinrich Stadial (HS), with darker grade shades indicating the late stage of HSs when BWT and SpSIC drop. Light gray shadings indicate Greenland Stadial (GS) (1). All data is presented on the GICC05modelext b2k timescale (2) (see Supporting Text S1).

**
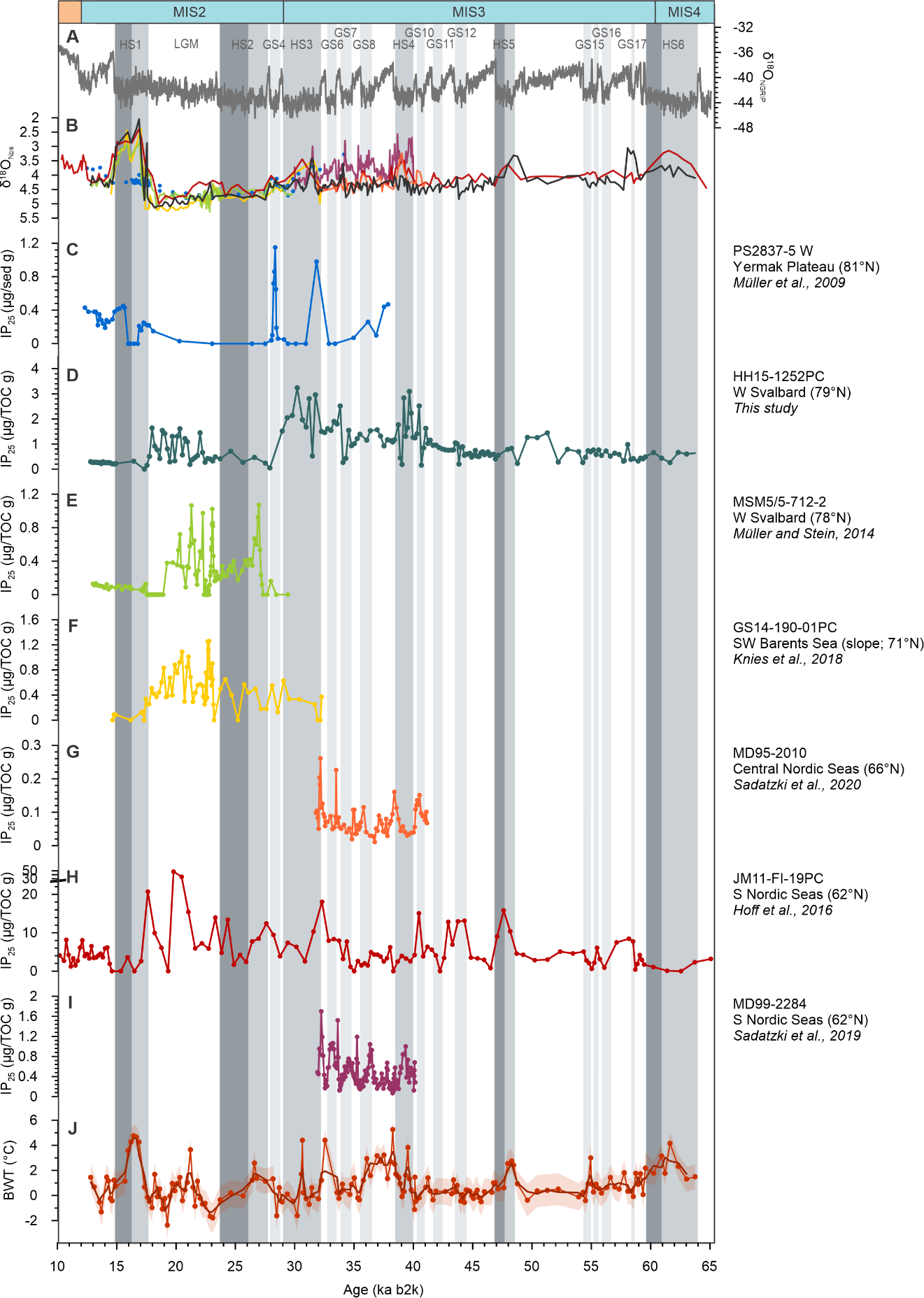
Fig. S5. Compilation of IP_25_ data from the south-east Nordic Seas to the Yermak Plateau.** (**A**) NGRIP ice-core δ^18^O on the GICC05modelext b2k timescale (2–4). (**B**) Planktic foraminiferal δ^18^O (δ^18^O_Nps_) of the different cores (**C–I**) IP_25_ recorded in cores from the literature and used in the discussion (see Table S1). (**J**) Reconstructed bottom water temperature (BWT) and its uncertainty interval (red shading) (1). Dark gray shadings mark Heinrich Stadial (HS), with darker grade shades indicating the late stage of HSs when BWT and SpSIC drop. Light gray shadings indicate Greenland Stadial (GS) (1). All data is presented on the GICC05modelext b2k timescale (2) (see Supporting Text S1).

Table S1. References for sea-ice biomarkers and planktic foraminiferal δ^18^O data from the south-east Nordic Seas to the Yermak Plateau used in the discussion. Planktic foraminiferal δ^18^O was used to correlate the records if needed (see Supporting text 1).

| **Core** | **Area** | **Time period (ky BP)** | **Type of data** | **Lat/Long** | **Reference** | **Dataset** | |
| --- | --- | --- | --- | --- | --- | --- | --- |
|  |  |  |  |  |  | **Biomarker** | **Planktic isotopes** |
| MD99-2284 | S Norwegian Sea (S Nordic Seas) | 32-41 | Brassicasterol, IP_25_ | 62.374667N  0.980167W | Sadatzki et al., 2019 (6) | Sadatzki et al. 2020 – suppl. Material (7) | Dokken et al., 2013 (8) |
| JM11-FI-19PC | S Norwegian Sea (S Nordic Seas) | 0-90 | Brassicasterol, Dinosterol, P_B_IP_25_, P_D_IP_25_, IP_25_ | 62.832830N  3.867170W | Hoff et al., 2016 (9)  Ezat et al., 2014 (10) | <https://doi.pangaea.de/10.1594/PANGAEA.859992> | <https://doi.pangaea.de/10.1594/PANGAEA.859992> |
| MD95-2010 | Central Norwegian Sea (S Nordic Seas) | 32-41 | Brassicasterol, HBI III, IP_25_ | 66.68N  4.57E | Sadatzki et al., 2020 (7) | Sadatzki et al. 2020 – suppl. Material (7) | <https://doi.org/10.1594/PANGAEA.61471> |
| GS14-190-01PC | SW Barents Sea (slope) | 16-27 | IP_25_ | 71.4755N  16.1650E | Knies et al., 2018 (11) | Knies et al., 2018 – suppl. material (11) | Knies et al., 2018 – suppl. material (11) |
| MSM5/5-712-2 | W Svalbard margin | 11-30 | Brassicasterol, P_B_IP_25_, P_D_IP_25_, IP_25_ | 78.915662N  6.767167E | Müller and Stein, 2014 (12) | <https://doi.pangaea.de/10.1594/PANGAEA.833668> | Zamelczyk et al., 2014 (13) |
| HH15-1252PC | W Svalbard margin | 13-64 | HBI III, IP_25_, SpSIC, HBI T_25_ | 79.041517N  6.887283E | This study | This study | <https://doi.org/10.1594/PANGAEA.925428> |
| PS93/006-1 | NW Barents Sea | 0-190 | IP_25_ | 79.203670N  4.668830E | Kremer et al., 2018 (14) | <https://doi.pangaea.de/10.1594/PANGAEA.884797> | Not correlated |
| PS92/039-2 | Yermak Plateau/Sofia Basin | 0-160 | Brassicasterol, HBI III, IP_25_ | 81.949830N  13.828330E | Kremer et al., 2018 (14) | <https://doi.pangaea.de/10.1594/PANGAEA.884792> | Not correlated |
| PS2837-5 | Yermak Plateau/Fram Strait | 0-30 | Brassicasterol, HBI III, IP_25_ | 81.233333N  2.381667E | Müller et al., 2009 (15) | <https://doi.org/10.1594/PANGAEA.728973> | <https://doi.org/10.1594/PANGAEA.107125> |

Table S2. Relative succession of events during stadials and at their transition with interstadials in the eastern Nordic Seas.

|  |  | **Relative timing of events** | | | | **References** |
| --- | --- | --- | --- | --- | --- | --- |
|  |  | **Early stadial** | **Mid/Late stadial** | **Late/End stadial** | **Early interstadial** |  |
| **NE Nordic Seas** | BWT | **↗** | **↘** | **-** | **-** | (1, 12, 16–19) |
|  | SST | **↘** |  | (**↗**?) | **↗** |  |
|  | Sea-ice cover | **↗** | **↘** | **-** |  |  |
|  | Ice-sheet retreat/Freshwater input |  |  | **↗ *** | **↗ *** |  |
|  | Atmospheric temperature | **- - -** | **- - -** | **- - -** | **+** |  |
| **Central Nordic Seas (E Norwegian Sea, Vøring Plateau)** | BWT | **?** | | | | (7, 17, 20–23) |
|  | SST | **↘** |  |  | **↗** |  |
|  | Sea-ice cover | **↗ **** |  | **↘ **** | **-** |  |
|  | Ice-sheet retreat/Freshwater input |  | **↗ *** | **↗ *** |  |  |
|  | Atmospheric temperature | **- -** | **- -** | **- -** | **+ +** |  |
| **SE Nordic Seas** | BWT | **↗** |  | **↘** | **-** | (6, 9, 10, 24–27) |
|  | SST | **↘** |  |  | **↗** |  |
|  | Sea-ice cover | **↗** | **↘** | **-** |  |  |
|  | Ice-sheet retreat/Freshwater input | **↗ *** | **↗ *** |  |  |  |
|  | Atmospheric temperature | **-** | **-** | **-** | **+ + +** |  |

↗ = increase; ↘ = decrease; - = low/lower (relatively to other regions in table); + = high/higher (relatively to other regions in table); * Short-term records (32 to 40 ka); **Known for the late deglaciation-HS1 (Lekens et al., 2006 includes HS 4, HS 3 and HS 2); Atmospheric temperature based on modern latitudinal differences. BWT=Bottom Water Temperature; SST=Sea (sub)Surface Temperature (planktic foraminifera-based)

**SI References**

1. N. El bani Altuna, M. M. Ezat, M. Greaves, T. L. Rasmussen, Millennial‐scale changes in bottom water temperature and water mass exchange through the Fram Strait 79^o^N, 63–13 ka. *Paleoceanogr. Paleoclimatol.* **36**, 21 (2021).

2. S. O. Rasmussen, *et al.*, A stratigraphic framework for abrupt climatic changes during the Last Glacial period based on three synchronized Greenland ice-core records: refining and extending the INTIMATE event stratigraphy. *Quat. Sci. Rev.* **106**, 14–28 (2014).

3. A. Svensson, *et al.*, A 60 000 year Greenland stratigraphic ice core chronology. *Clim. Past.* **4**, 47–57 (2008).

4. E. W. Wolff, J. Chappellaz, T. Blunier, S. O. Rasmussen, A. Svensson, Millennial-scale variability during the last glacial: The ice core record. *Quat. Sci. Rev.* **29**, 2828–2838 (2010).

5. S. P. Jessen, T. L. Rasmussen, Ice-rafting patterns on the western Svalbard slope 74-0 ka: interplay between ice-sheet activity, climate and ocean circulation. *Boreas* **48**, 236–256 (2019).

6. H. Sadatzki, *et al.*, Sea ice variability in the southern Norwegian Sea during glacial Dansgaard-Oeschger climate cycles. *Sci. Adv.* **5**, eaau6174 (2019).

7. H. Sadatzki, *et al.*, Rapid reductions and millennial-scale variability in Nordic Seas sea ice cover during abrupt glacial climate changes. *Proc. Natl. Acad. Sci.* **117**, 29478–29486 (2020).

8. T. M. Dokken, K. H. Nisancioglu, C. Li, D. S. Battisti, C. Kissel, Dansgaard-Oeschger cycles: Interactions between ocean and sea ice intrinsic to the Nordic seas. *Paleoceanography* **28**, 491–502 (2013).

9. U. Hoff, T. L. Rasmussen, R. Stein, M. M. Ezat, K. Fahl, Sea ice and millennial-scale climate variability in the Nordic seas 90 kyr ago to present. *Nat. Commun.* **7**, 12247 (2016).

10. M. M. Ezat, T. L. Rasmussen, J. Groeneveld, Persistent intermediate water warming during cold stadials in the southeastern Nordic seas during the past 65 k.y. *Geology* **42**, 663–666 (2014).

11. J. Knies, *et al.*, Nordic Seas polynyas and their role in preconditioning marine productivity during the Last Glacial Maximum. *Nat. Commun.* **9**, 3959 (2018).

12. J. Müller, R. Stein, High-resolution record of late glacial and deglacial sea ice changes in Fram Strait corroborates ice–ocean interactions during abrupt climate shifts. *Earth Planet. Sci. Lett.* **403**, 446–455 (2014).

13. K. Zamelczyk, T. L. Rasmussen, K. Husum, F. Godtliebsen, M. Hald, Surface water conditions and calcium carbonate preservation in the Fram Strait during marine isotope stage 2, 28.8-15.4 kyr. *Paleoceanography* **29**, 1–12 (2014).

14. A. Kremer, *et al.*, Changes in sea ice cover and ice sheet extent at the Yermak Plateau during the last 160 ka – Reconstructions from biomarker records. *Quat. Sci. Rev.* **182**, 93–108 (2018).

15. J. Müller, G. Massé, R. Stein, S. T. Belt, Variability of sea-ice conditions in the Fram Strait over the past 30,000 years. *Nature Geosci.* **2**, 772–776 (2009).

16. Rasmussen, T. L., Thomsen, E. & Nielsen, T. Water mass exchange between the Nordic seas and the Arctic Ocean on millennial timescale during MIS 4-MIS 2. *Geochemistry, Geophysics, Geosystems* **15**, 530–544 (2014).

17. T. L. Rasmussen, E. Thomsen, Pink marine sediments reveal rapid ice melt and Arctic meltwater discharge during Dansgaard–Oeschger warmings. *Nature Communications* **4** (2013).

18. A. L. C. Hughes, R. Gyllencreutz, Ø. S. Lohne, J. Mangerud, J. I. Svendsen, The last Eurasian ice sheets - a chronological database and time-slice reconstruction, DATED-1. *Boreas* **45**, 1–45 (2016).

19. H. Patton, *et al.*, Deglaciation of the Eurasian ice sheet complex. *Quat. Sci. Rev.* **169**, 148–172 (2017).

20. Dokken, T. M. & Jansen, E. Rapid changes in the mechanism of ocean convection during the last glacial period. *Nature* **401**, 458–461 (1999).

21. W. A. H. Lekens, *et al.*, Laminated sediments preceding Heinrich event 1 in the Northern North Sea and Southern Norwegian Sea: Origin, processes and regional linkage. *Marine Geology* **216**, 27–50 (2005).

22. B. Hjelstuen, *et al.*, Late Quaternary seismic stratigraphy and geological development of the south Vøring margin, Norwegian Sea. *Quat. Sci. Rev.* **23**, 1847–1865 (2004).

23. B. O. Hjelstuen, *et al.*, Late Cenozoic glacial history and evolution of the Storegga Slide area and adjacent slide flank regions, Norwegian continental margin. *Mar. Pet. Geol.* **22**, 57–69 (2005).

24. Wary, M. *et al.* Regional seesaw between the North Atlantic and Nordic Seas during the last glacial abrupt climate events. *Clim. Past* **13**, 729–739 (2017).

25. E. G. Sessford, *et al.*, Consistent fluctuations in intermediate water temperature off the coast of Greenland and Norway during Dansgaard-Oeschger events. *Quat. Sci. Rev.* **223**, 105887 (2019).

26. W. A. H. Lekens, H. P. Sejrup, H. Haflidason, J. Knies, T. Richter, Meltwater and ice rafting in the southern Norwegian Sea between 20 and 40 calendar kyr B.P.: Implications for Fennoscandian Heinrich events. *Paleoceanography* **21** (2006).

27. S. Toucanne, *et al.*, Millennial-scale fluctuations of the European Ice Sheet at the end of the last glacial, and their potential impact on global climate. *Quat. Sci. Rev.* **123**, 113–133 (2015).
